# Characterization of the complete mitochondrial genome of *Lymantria sugii* from China and its phylogenetic analysis

**DOI:** 10.1080/23802359.2020.1778563

**Published:** 2020-06-16

**Authors:** Zhi-yi Wu, Tao Wang, Peng-cheng Chen, Fan Cheng, Hong-wei Tian, Yan Ren

**Affiliations:** a Zhejiang Academy of Science and Technology for Inspection and Quarantine, Hangzhou, PR China; b Guizhou Light Industry Technical College, Guiyang, PR China

**Keywords:** Mitogenome, Lymantriinae;·*Lymantria sugii*, phylogeny

## Abstract

The complete mitochondrial genome (mitogenome) of *Lymantria sugii* (Diptera: Tephritidae: Dacinae) was sequenced and annotated. The mitochondrial genome is 15,614 bp (GenBank No. MT265380), containing 80.4% A + T (A 39.1%, C 7.3%, G 12.2%, and T 41.3%), that is heavily biased toward A and T nucleotides. All PCGs were started with ATN (ATA/ATG/ATT/ATC) and were terminated with TAR (TAA/TAG) excepting ND4, which ended with single T. Additionally, the phylogenetic tree confirmed that *L. sugii* clustered with *L. umbrosa*, *L. dispar* and *Lymantria* sp. The current study would be enrich the mitogenomes of the Lymantriinae.

*Lymantria sugii* belongs to *Lymantria* genus. Many species in *Lymantria* genus are known as significant forestry pests. Currently, the *Lymantria* genus includes 173 species, containing 12 confirmed subgenera all over the world. Among them, 31 species of 3 subgenera were selectively reviewed as potential invaders to North America (Kang et al. 2015). The identification of *Lymantria sugii* is mainly based on morphology and partial sequence. Presently, we have sequenced and determined the complete mitochondrial genome (mitogenome) using next-generation sequencing method for the first time, which might facilitate future studies on the Lymantriinae.

The genome DNA was extracted from male adult of *Lymantria sugii* which was collected in Zhuanghe area, Dalian city, China (E 123°10′43.79″, N 39°42′14.34″), in September 2019, and the voucher specimen are deposited in the Insect Collection of Zhejiang Academy of Science & Technology for Inspection & Quarantine (ZAIQ) with label number ZAIQ-LL-SM-1901. These sequences were assembled using Geneious Primer (Kearse et al. 2012), version 10.2.3 (http://www.geneious.com/). Additionally, all tRNAs were found by MITOS server (http://mitos.bioinf.uni-leipzig.de/index.py) (Bernt et al. 2013) and tRNA scan-SE server (Lowe and Chan 2016) for annotation. The neighbor-joining (NJ) tree was constructed to investigate the molecular taxonomic position of *Lymantria sugii* basing on nucleotide sequences of 13 protein-coding genes and 2 rRNA genes using MEGA 6.0 (Tamura et al. 2013) from alignments created by the MAFFT (Katoh and Standley 2013).

The complete mitogenome of *Lymantria sugii* is 15,614 bp (GenBank No. MT265380), containing 22 transfer RNA genes (tRNAs, 1470 bp), 13 protein-coding genes (PCGs, 11,074 bp), 2 ribosomal RNA genes (rRNAs, 2181 bp), and one non-coding region (Control region, 391 bp). The whole genome contained 39.1% A, 7.3% C, 12.2% G, and 41.3%T, showing an obvious A + T bias (80.4%). The AT-skew and GC-skew for the whole mitogenome is –0.0274 and 0.2513 respectively. All PCGs were started with ATN (ATA/ATG/ATT/ATC) and terminated with TAR (TAA/TAG) excepting ND4, which ends with single T.

The phylogenetic relationships of *L. sugii* were reconstructed using the neighbor-joining (NJ) method with 1000 bootstrap replicates basing on concatenated nucleotides of the 13 PCGs and 2 rRNAs with 13,194 bp, *Dosophila suzukii and Drosophila melanogaster* were used as outgroup (Figure 1). The phylogenetic tree confirmed that *L. sugii* clustered with *L. dispar*, *Lymantria* sp. and *L. umbrosa.* Presently, the studies recording *L. sugii* were limited, and we believe that our data could be useful for further study.

**Figure 1.**
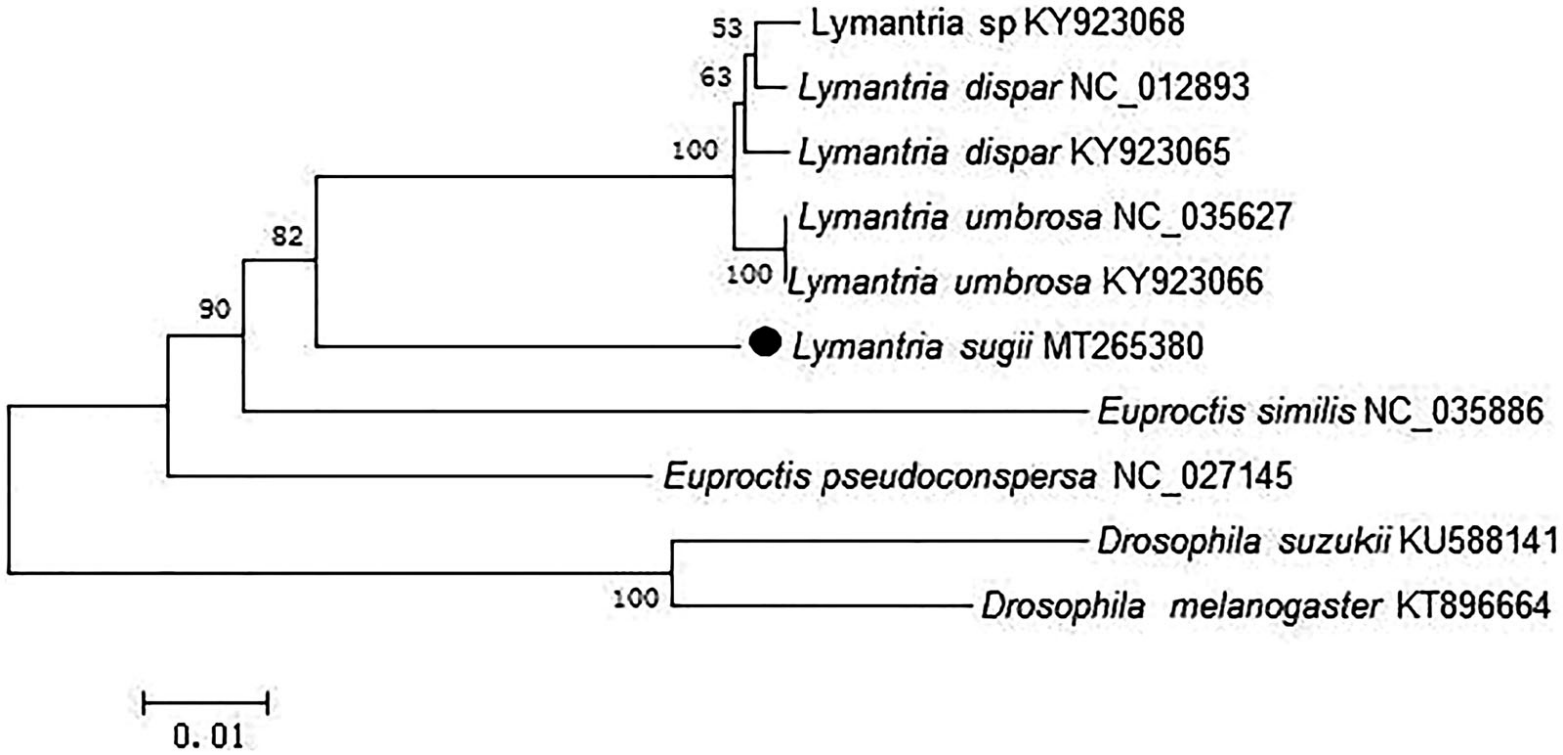
Neighbor-joining (NJ) phylogenetic tree of *L. sugii* basing on concatenated nucleotides of the 13 PCGs and 2 rRNAs by MEGA 6.0.

## Data Availability

The data that support the findings of this study are openly available in NCBI at https://www.ncbi.nlm.nih.gov/, reference number MT265380.
